# MLPA for confirmation of array CGH results and determination of inheritance

**DOI:** 10.1186/1755-8166-3-19

**Published:** 2010-10-13

**Authors:** Alison Hills, Joo Wook Ahn, Celia Donaghue, Helen Thomas, Kathy Mann, Caroline Mackie Ogilvie

**Affiliations:** 1 Cytogenetics Department, GSTS Pathology, London SE1 9RT, UK; 2 Cytogenetics Department, Guy's & St Thomas' NHS Foundation Trust, London SE1 9RT, UK

## Abstract

**Background:**

Array CGH has recently been introduced into our laboratory in place of karyotype analysis for patients with suspected genomic imbalance. Results require confirmation to check sample identity, and analysis of parental samples to determine inheritance and thus assess the clinical significance of the abnormality. Here we describe an MLPA-based strategy for the follow-up of abnormal aCGH results.

**Results:**

In the first 17 months of our MLPA-based aCGH follow-up service, 317 different custom MLPA probes for novel aCGH-detected abnormalities were developed for inheritance studies in 164 families. In addition, 110 samples were tested for confirmation of aCGH-detected abnormalities in common syndromic or subtelomeric regions using commercial MLPA kits. Overall, a total of 1215 samples have been tested by MLPA. A total of 72 de novo abnormalities were confirmed.

**Conclusions:**

Confirmation of aCGH-detected abnormalities and inheritance of these abnormalities are essential for accurate diagnosis and interpretation of aCGH results. The development of a new service utilising custom made MLPA probes and commercial MLPA kits for follow-up studies of array CGH results has been found to be efficient and flexible in our laboratory.

## Background

Array Comparative Genomic Hybridisation (aCGH) using either Bacterial Artificial Chromosome (BAC) or oligonucleotide platforms is currently the method of choice for genome-wide screening for chromosome imbalance [1-6]. This technique is now widely used in cytogenetic laboratories as a follow-up test for patients with phenotype suggestive of chromosome imbalance, but with normal karyotypes. An international consensus statement has recently recommended array CGH as a first-line test in place of traditional karyotype analysis [7]; implementation of this recommendation requires that any aCGH diagnostic service should be robust, cost-effective and medium-throughput.

An essential component of such a service is a strategy for confirming abnormal findings, and for establishing the carrier status of the parents. Various strategies for confirmation of abnormal aCGH findings have been described, including repeat aCGH testing [8], Fluorescence In Situ Hybridization (FISH), microsatellite analysis [9,10], real-time PCR [11,12] and Multiplex Ligation-dependent Probe Amplification (MLPA) for specific genes [8]. Some centres confirm abnormal findings only for small imbalances, due to the confidence associated with abnormal "calls" on multiple adjacent probes (personal communication), whereas others confirm all abnormal findings [1,2]

Inheritance information is essential for accurate interpretation of aCGH findings, and the above strategies can also be applied for testing parental DNA [12-14].

In our laboratory, we have recently introduced oligonucleotide aCGH in place of karyotype analysis [15]. In this paper we describe an MLPA-based approach for inheritance studies, and for confirmation of aCGH abnormalities within common syndromic regions (e.g. chromosome 22q11 microdeletion region) and subtelomeric regions. Our strategy utilises both custom-designed MLPA probes and commercially available MLPA kits used in our existing diagnostic service for products of conception [16] This approach results in considerable time and efficiency savings for our aCGH service.

## Methods

### Array Comparative Genomic Hybridisation

Array CGH analysis was carried out as described previously [15]. Briefly, DNA was labelled using CGH Labelling Kit for Oligo Arrays (Enzo Life Sciences, USA), then applied to 44 k oligonucleotide arrays (Agilent, USA). Image quantification, array quality control and aberration detection were performed using Feature Extraction and DNA Analytics software packages (Agilent, USA) according to the manufacturer's instructions.

### DNA preparation

All DNA samples for MLPA testing were prepared and quality tested as described previously [17,18]. DNA was quantified by spectrophotometry (Nanodrop, USA) and checked for degradation on an agarose gel. Degraded DNA was not used; new samples were requested for these cases.

### MLPA custom probe design and synthesis

Custom MLPA probes were designed online using the publicly available Human MLPA Probe Design algorithm (H-MAPD; http://genomics01.arcan.stonybrook.edu/mlpa/cgi-bin/mlpa.cgi) [19]. Probes of various sizes were designed by specifying the required length of ligation product, with no stuffer sequence. The suggested probe sequences were subjected to BLAT searches http://genome.ucsc.edu/ to verify genomic position and sequence homology. Right hybridizing sequences (RHS) were ordered as 5' phosphorylated "Ultramer"oligonucleotides and left hybridizing sequences (LHS) were ordered as standard desalted oligonucleotides (Integrated DNA Technologies, Belgium).

### Custom MLPA assay selection

MRC-Holland manufacture two MLPA kits, P200 and P300, designed for use with custom MLPA probes. These kits combine reference probes for data normalization and quantity/quality control fragments with space for the addition of multiple custom probes. Both kits were trialled and a final decision made to routinely use the P200 kit as it allowed shorter and therefore cheaper custom probes to be used than the P300 kit. The P300 kit is preferred by users who observe significant effects of signal sloping on their data and counters this problem by distributing reference probes over the whole size range of the probemix. This signal sloping effect was not observed to a significant degree with our data.

**Figure 1:**
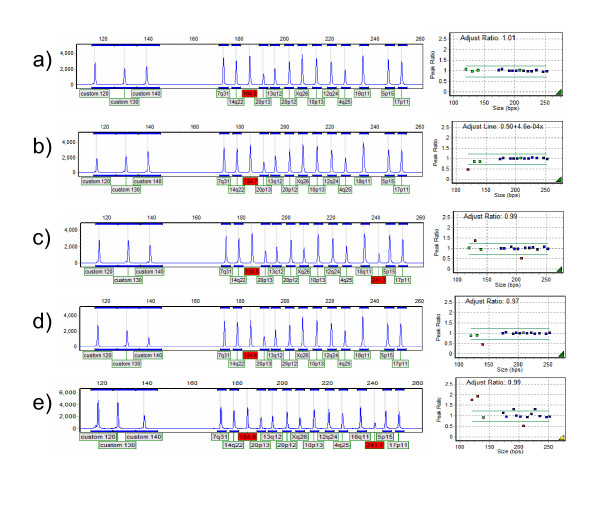
**MLPA results of three custom probes in the MRC-Holland P200 kit**. GeneMarker electrophoretograms are shown on the left, normalized MLPA data on the right. The 184 bp peak represents the D-fragment which is used to highlight incomplete denaturation and is not shown in the analysed data on the right, the 241 bp fragment is a Y chromosome specific fragment for the identification of male samples. a) normal female control sample b) female sample with decreased copy number for the 120 bp probe c) male sample with increased copy number for the 130 bp probe d) female sample with decreased copy number for the 140 bp probe e) poor quality male sample with several outlying probes.

Up to three custom MLPA probes, differing in size by at least ten base pairs, were run concurrently in a single probe mix (see Figure 1). This reduced costs by reducing the number of normal control reactions and the associated consumables needed. The potential for making efficiency and consumable savings by increasing the number of probes per mix must be weighted against the cost of probes of increasing size. In our laboratory the most efficient scenario involves the use of three separate four probe mixes, with three family members tested per probe. This allows 12 families to be tested per 96 well plate.

### Multiplex Ligation-dependent Probe Amplification

MLPA reactions using MRC-Holland SALSA^® ^P245, P36E and P69 kits were carried out according to the manufacturer's instructions and as described previously [17].

Custom MLPA probes were prepared and diluted and reactions performed according to the synthetic MLPA protocol provided on the MRC-Holland website http://mlpa.com.

No optimisation of custom probe concentration was required. Peak heights were found to be generally comparable to those of the P200 reference probes when probes were added at the concentrations recommended by MRC-Holland.

DNA samples tested with custom probes were tested in duplicate; two normal control samples, each tested in triplicate, and a negative control were included for every custom probe mix. The proband was always tested alongside parental samples to confirm assay sensitivity.

### MLPA data analysis

A 3 μl aliquot of PCR product was mixed with 0.3 μl Genescan 500 LIZ size standard (Applied Biosystems, UK) and 15 μl HiDi formamide (Applied Biosystems, UK) before being size-separated by capillary electrophoresis on a 3730 genetic analyser (Applied Biosystems, UK) according to the manufacturer's instructions.

MLPA peak height data was analysed using GeneMarker v1.8 software (SoftGenetics, USA). A maximum raw data peak height of 15,000 relative fluorescent units (rfu) was established after observing a loss of peak height relative dosage with increasing concentrations of DNA. Peak heights which were too low also produced unreliable dosage ratios; a lower peak height limit was set at 250 rfu. Results with peak heights outside of these limits were failed and the sample re-processed.

#### P36, P69 and P245 MLPA kits

Data from the P36E and P69 subtelomere kits and the P245 microdeletion/microduplication kit were interpreted according to analysis parameters described previously [18].

#### Custom MLPA

Peak height data from MRC-Holland P200 kits with added custom probes were analysed according to recommendations on data normalization provided on the MRC-Holland website. Panels were designed to detect one or more custom probes per kit.

The internal control normalization function was selected so that sample data was only compared to user-specified control data rather than to the whole run. Custom probe peaks were not included in the normalization

The MLPA Analysis function in GeneMarker was used for determining copy number of custom probes with MLPA peak height ratios defined as deletion < 0.71 ≤ normal ≤ 1.23 ≤ duplication (as described by [17]). Figure 1 shows an example trace.

For a result to be called, at least one out of three replicates for each control sample must pass (i.e. peak height within limits and no outlying probe peaks), and both duplicate patient results must be concordant. Samples with reference probe peak ratios which fell outside of the normal range were failed (see Figure 1e), as were samples with high QC fragment peaks.

This analysis approach is similar to that used for our diagnostic service for the molecular analysis of miscarriage products [16]

### Genotyping

Genotyping for relationship testing (in families where the abnormality in the proband had apparently arisen de novo) was carried out using multiplex quantitative fluorescence PCR (QF-PCR) assays containing multiple polymorphic STR markers which are routinely used in our department for aneuploidy diagnosis [20,21].

## Results

In the period from January 2009 to May 2010, 3306 array CGH tests were carried out in our laboratory. The following sections refer to MLPA results obtained within this time period

### Confirmation of array CGH results and inheritance studies using custom MLPA probes

When parental blood samples were received for follow-up studies of aCGH findings not covered by probes in MRC-Holland P36, P69 or P245 kits, a custom MLPA probe specific for a locus within the abnormal region was designed. Potentially relevant genes were targeted and common copy number variants (CNV) were avoided.

**Figure 2:**
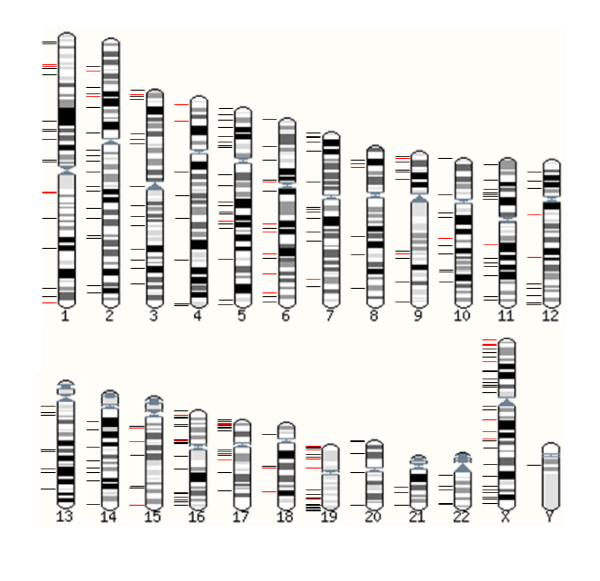
**Karyotype illustrating the spread of custom probes across the genome**. Black bars indicate that a probe has been tested on one family, red bars indicate that a probe has been tested on more than one family.

During this period, 317 unique custom MLPA probes were designed for 164 families in order to determine inheritance of both duplication and deletion results, and were tested on a total of 1105 individuals. Figure 2 depicts the spread of custom probes across the genome.

The majority of custom probes were used for only single families; however, certain regions have started to emerge as exhibiting recurrent deletions and/or duplications. This may be due to previously unidentified recurrent syndromic regions, or may represent benign population polymorphisms. To date, the probe with the greatest usage is located at Xp22.31. This has been used for 11 families. Duplication of this region has been found in patients with a range of different phenotypes, and also in normal parents, both male and female, and has therefore been suggested as a benign variant [22]. The reciprocal deletion gives rise to steroid sulphatase deficiency in male carriers (OMIM 306480).

In addition, a custom probe has been designed for the autism susceptibility locus on chromosome 16p11.2 [23-25]. Copy number changes within this region are frequently detected by aCGH and are very likely to arise following unequal meiotic crossing over between flanking low-copy repeats. Custom MLPA can therefore be used to confirm the abnormality, thus eliminating the need for repeat aCGH testing in such cases. Within our data set, this custom MLPA probe has been used to confirm copy number changes and for inheritance studies in five families.

Twenty seven custom MLPA probes (8.5%) failed, due to either amplification failure or inconsistent results. In all cases, replacement probes were designed.

### Confirmation of subtelomeric abnormalities and common microdeletion/microduplications detected by aCGH

During this period, 49 samples from 28 families were tested using subtelomere kits (P36E and P69) for follow-up of aCGH results. Three de novo terminal abnormalities were confirmed out of a total of nine where inheritance studies have been completed; a chromosome 9p deletion, a proximal chromosome 13q deletion (confirmed using the P36E kit which contains a probe for the proximal region of the long arm of chromosome 13) and an unbalanced translocation between the telomeric regions of the short arms of chromosomes 5 and 8.

In the same period, 61 samples from 32 families were tested using microdeletion/microduplication kits (P245) for follow-up of aCGH results. Eleven de novo results were confirmed out of a total of twelve where inheritance studies had been completed; six deletions of the chromosome 22q11 microdeletion/microduplication region, three abnormalities in the Prader Willi/Angelman syndrome region, one duplication of the Smith-Magenis region, one partial deletion of the Miller-Dieker region, one deletion of the NF1 region, one deletion of the Alagille region and one duplication of the Williams Beuren region. One case of an inherited chromosome 22q11 duplication was also confirmed.

### Discrepant results

We observed no discrepancies between array CGH results and MLPA results. Apparent discrepancies may arise due to SNPs close to the ligation site of the two probe hybridising sequences, causing amplification failure, and therefore mimicking a deleted result. This is mitigated by using the H-MAPD software which checks potential probe sequences against the latest SNP database.

### Results turnaround times

Confirmation and inheritance results were generally available within two weeks of sample receipt. Probes were usually synthesized and dispatched by IDT within five working days of ordering. Tests using existing stocks of probes could be turned around in less than a week in urgent cases. The rate of repeat testing due poor DNA quality or discordant probe results was 14%.

## Discussion

Array CGH testing for genomic imbalance has been introduced as a first-line test in place of G-banded chromosome analysis in a number of centres, and is offered as an additional test by most cytogenetics laboratories. As with any test protocol that involves the simultaneous handling of multiple DNA samples, it is important that any abnormal results are confirmed in order to establish patient identity before reporting. However, repeat array CGH testing of all abnormal findings, and array testing of parental samples for inheritance studies adds significantly to the cost of the service and may also result in incidental findings potentially requiring further investigation.

MLPA is now an established technique for the investigation of copy number [26]. MRC-Holland http://www.mrc-holland.com manufactures numerous commercial MLPA kits for the detection of copy number changes in different genomic locations. We currently use three of these kits (P36E, P69 and P245) in our recently described diagnostic service for the molecular analysis of miscarriage products [16]. Prior to the introduction of aCGH into our laboratory we also used these kits for the diagnosis of subtelomeric imbalance (P36E and P69) and common microdeletion/microduplication syndromes (P245) in patients with idiopathic developmental delay and/or dysmorphism [17]. We therefore have in operation in our laboratory an efficient and medium throughput diagnostic MLPA-based service and have refined our sample preparation and data analysis techniques to improve accuracy and turnaround times. We were able to exploit this existing service in order to develop an efficient follow-up strategy for aCGH testing. However, the approach we describe should also be feasible in centres with an appropriate genetic analyser without previous experience of this technique, as extensive technical support is available from MRC-Holland, and DNA preparation, probe design, reaction set up and data analysis protocols are readily available. In our experience, optimisation is not required for the majority of custom MLPA probes. Furthermore, the use of custom MLPA probes represents a simple method for establishing copy number beyond the usage described in this paper.

This approach results in significant time and cost savings by avoiding the need for aCGH testing for confirmation of common abnormalities or of parental samples. Other follow-up approaches that have been described include FISH, which has the advantage of giving position information, and excluding balanced rearrangements in parents, in cases of apparent de novo findings [13]. However, this approach requires cell culture in order to examine chromosome spreads, whereas MLPA is performed on the same DNA sample as is used for the original aCGH analysis; in addition some regions of imbalance detected by aCGH are too small to be followed up by routinely-available FISH probes. Nevertheless, where FISH probes are available, we offer FISH testing to parents of children with de novo abnormalities, in order to refine their reproductive risk; no families have yet requested this testing. We have compared real-time PCR as a follow-up strategy and have found it to be more costly and time-consuming than MLPA (results not shown).

Follow-up of aCGH results using STR markers and QF-PCR is limited by a lack of polymorphic markers, time taken for optimization and the inability to confirm deletions in the absence of parental genotypes. However, we are able to confirm common trisomies using the multiplex QF-PCR kits designed for our prenatal service [20,27].

## Conclusions

Follow-up of abnormal aCGH results is good practice to confirm patient identity, and inheritance studies are necessary for accurate interpretation of these abnormal findings. The results presented here demonstrate that MLPA is a fast, robust and accurate technique for confirmation and follow-up of both duplication and deletion aCGH results. However, exclusion of balanced parental rearrangements by FISH is recommended for apparently de novo abnormalities.

## Competing interests

The authors declare that they have no competing interests.

## Authors' contributions

AH developed the custom MLPA probe approach, designed the probes, analysed the data and wrote the initial draft of the paper. JWA developed the MLPA diagnostic analysis strategy and helped with the data analysis for the paper. CD helped to develop the custom MLPA probe approach and design the custom MLPA probes, and carried out the diagnostic interpretation. HT carried out the routine testing and assisted with interpretation. KM initiated MLPA testing in our laboratory, helped with development of the MLPA diagnostic analysis strategy, and advised on the development of this service. CMO initiated and led the development of this service, and co-wrote the paper.

All the authors have provided input for the paper and have read and approved the final manuscript.
